# Intervention With *Lacticaseibacillus paracasei* PC‐01 Fermented Milk Beverage Ameliorates Functional Dyspepsia and Modulates Gut Microbiome: A Pilot Study

**DOI:** 10.1002/fsn3.71928

**Published:** 2026-07-01

**Authors:** Xiangyang Zhang, Erna Sun, Zhixin Zhao, Shusen Li, Xin Shen, Julong Liu, Qiuwen He, Yuejiao Wang, Feiyan Zhao, Hongfeng Zhao, Heping Zhang

**Affiliations:** ^1^ Key Laboratory of Dairy Products Processing, Ministry of Agriculture and Rural Affairs Inner Mongolia Agricultural University Hohhot China; ^2^ Key Laboratory of Dairy Biotechnology and Engineering, Ministry of Education Inner Mongolia Agricultural University Hohhot China; ^3^ Inner Mongolia Key Laboratory of Dairy Biotechnology and Engineering Inner Mongolia Agricultural University Hohhot China; ^4^ Mengniu Hi‐Tech Dairy Product Beijing co., Ltd. Beijing China; ^5^ Wuhan Business University Wuhan China

**Keywords:** functional dyspepsia, gastrointestinal symptom rating scale, global overall symptom scale, gut microbiota, *Lacticaseibacillus paracasei* PC‐01

## Abstract

Functional dyspepsia (FD) is a common chronic gastrointestinal disorder characterized by persistent or recurrent epigastric symptoms in the absence of detectable structural abnormalities. In this pilot study, we explored whether a *Lacticaseibacillus paracasei* PC‐01 (PC‐01) fermented milk beverage alleviates FD symptoms. Fifty‐five patients with FD were randomized into an experimental group (EP, *n* = 37) receiving the PC‐01 fermented milk beverage (5.0 × 10^8^ CFU/mL, 200 mL/day) or a control group (CP, *n* = 18) receiving the active comparator, an acidified milk beverage (non‐fermented, without PC‐01) (200 mL/day). The interventions lasted 28 days, with symptom scores on the 7‐point Global Overall Symptom Scale (GOSS) and Gastrointestinal Symptom Rating Scale (GSRS), and fecal samples were collected at baseline (day 0), 14, and 28. Consumption of the PC‐01 fermented milk beverage in this pilot study was associated with improvements in FD symptoms, and a higher effective response rate was observed in the EP group than in the CP group (*p* = 0.04). Metagenomic analysis revealed that, compared with the CP group, the EP group exhibited significant enrichment of potentially beneficial bacteria (e.g., *Blautia*) and a reduction in potentially pathogenic bacteria (e.g., *Clostridium paraputrificum* ), accompanied by significant downregulation of the fatty acid β‐oxidation I (FAO‐PWY) pathway. We acknowledge that the limitation of this pilot study is that the acidified milk beverage used as the control might also exert certain effects on gastrointestinal symptoms and gut microbiota, which could not be fully avoided due to the lack of a fully inert placebo. Collectively, the findings of this preliminary study indicate that the PC‐01 fermented milk beverage may alleviate FD‐related symptoms and modulate the gut microbiome and metabolic pathways, highlighting its potential in ameliorating FD‐associated symptoms. Further large‐sample, multi‐center, and long‐term clinical studies are warranted to verify these preliminary results and establish the long‐term efficacy and safety of FD management.

## Graphical abstract

This study confirmed that intake of the *Lacticaseibacillus paracasei* PC‐01 fermented milk beverage can significantly alleviate symptoms in patients with functional dyspepsia. It markedly increases the abundances of *Lacticaseibacillus paracasei* and *Blautia*, while reducing the abundance of *Enterobacter roggenkampi.* Meanwhile, the expression level of the Formaldehyde oxidation I (RUMP‐PWY) pathway is upregulated, whereas the pathways of Fatty acid β‐oxidation I (FAO‐PWY) and Ubiquinol‐7 biosynthesis (PWY‐5855) are downregulated. Therefore, the PC‐01 fermented milk beverage has the potential to serve as a non‐pharmacological intervention strategy for functional dyspepsia.

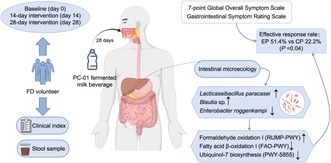

## 1 Introduction

Functional dyspepsia (FD) is a common gastrointestinal disorder characterized by persistent or recurrent symptoms, with high clinical morbidity and low cure rates (Xv et al. 2024). It is defined as the absence of observable organic lesions in the digestive tract. However, it presents with clinical symptoms such as postprandial discomfort, early satiety, and upper abdominal pain or burning sensation (Zhang et al. 2024). Western pharmacotherapy commonly employed for FD includes prokinetic agents, acid inhibitors, neuromodulators, and leukotriene receptor antagonists. However, long‐term use of these medications can induce adverse reactions (e.g., diarrhea, dizziness, vomiting, and skin rashes) and serious complications (e.g., headache, atrophic gastritis, and gastric polyps) (Zhao, Cheng, et al. 2024). Moreover, such treatment regimens impose additional economic and psychological burdens on patients and increase the risk of drug dependence and safety concerns (Ombasa et al. 2025). Therefore, FD requires urgent attention and the development of more effective intervention strategies.

In recent years, the gut microbiota has attracted increasing attention for its potential role in ameliorating gastrointestinal disorders. The gut microbiota contributes to nutrient absorption, immune system maturation, and host defense against intestinal bacterial infections. Moreover, it helps maintain host homeostasis by participating in metabolism and immune regulation (Rai et al. 2025). In a healthy state, the microbiota and host exist in a mutually interdependent state, maintaining a dynamic equilibrium in the microbial composition and abundance within the gut. However, disruption of this balance can lead to intestinal dysbiosis, characterized by the overgrowth of pathogenic bacteria, and may trigger a cascade of pathological changes (Abeltino et al. 2024). Gut microbiota dysbiosis has been identified as an important pathogenic mechanism of FD (Tziatzios et al. 2025). As research in this field has deepened, the association between the gut microbiota and FD has become increasingly evident. Many gastrointestinal diseases, including FD, are associated with gut microbial community imbalances. For example, Zhong et al. reported a significant increase in the abundance of *Streptococcus* in the duodenal mucosa of patients with FD compared to healthy controls, accompanied by a reduction in *Prevotella*, *Veillonella*, and *Actinomyces* (Zhong et al. 2017). In a rat model of FD with liver depression‐spleen deficiency syndrome, Qiu et al. observed a decreased relative abundance of *Bacteroidetes* and increased abundances of *Firmicutes*, *Proteobacteria*, and *Cyanobacteria* (Qiu et al. 2017). These findings suggest that gut microbiota dysregulation may be a key mechanism underlying the pathogenesis of FD.

Studies have shown that fermented foods, especially dairy products such as yogurt and fermented milk, which are rich in live active probiotics, provide various health benefits (Şanlier et al. 2019). Many studies have reported that these products can improve the symptoms of gastrointestinal disorders by modulating the composition and function of intestinal flora, and they can also help enhance intestinal health in healthy individuals. For example, the intake of a multi‐strain probiotic yogurt increased the abundance of *Bifidobacterium* and *Sutterella* and decreased that of *Lachnoclostridium* and *Coprobacter* in the gut microbiota of patients with slow‐transit constipation, which coincided with improvements in constipation symptoms (XiaoRan et al. 2020). Fermented milk containing *Lacticaseibacillus casei* Zhang ( *L. casei* Zhang) and *Bifidobacterium animalis* ssp. *lactis* V9 ( *B. animalis* ssp. *lactis* V9) increased the abundance of *Streptococcus thermophilus* ( *S. thermophilus* ) and *B. animalis* in the gut, along with improved defecation frequency and amelioration of constipation symptoms (Wang et al. 2020). In addition, the consumption of a fermented milk beverage containing *B. animalis* significantly improved constipation‐related symptoms in patients (Moreira et al. 2017). Collectively, these findings demonstrate that probiotic fermented products can modulate the gut microbiota and alleviate gastrointestinal symptoms in multiple disorders, suggesting their potential applicability in the treatment of FD.

The *L. paracasei* PC‐01 strain used in this study exhibited exceptional gastrointestinal tolerance, enabling it to survive passage through the digestive tract and effectively colonize the intestinal microbiota in an active state. It also has a favorable safety profile and demonstrates probiotic benefits (Kailong et al. 2021). Based on preliminary experimental data, PC‐01 exhibited a survival rate of 99.9% after 1 h incubation in simulated gastric fluid at pH 2.5 (10% MRS containing 3.2 mg/mL pepsin and 34 mM sodium chloride, adjusted to pH 2.5 with hydrochloric acid). Furthermore, PC‐01 has been shown to maintain robust survival and stability in milk beverages (Zhe et al. 2021). Given these advantageous characteristics and the known pathophysiology of FD, we hypothesized that PC‐01‐enriched dairy products could alleviate FD symptoms. Using commercial fermented milk beverages containing PC‐01 as an intervention ensured effective delivery of the probiotic strain and mimicked real‐world dietary consumption, facilitating the evaluation of its health effects in a practical dietary context. This approach is expected to improve participant acceptance and compliance, thereby enhancing the feasibility of the intervention. In the present study, we systematically investigated the impact of PC‐01 fermented milk beverages on patients with FD by analyzing their responses to questionnaires, gut microbiota composition, and microbial metabolic pathways. This study aimed to provide novel insights and references for dietary management and non‐pharmacological interventions in patients with FD.

## 2 Materials and Methods

### 2.1 Ethics Statement

This study was conducted in accordance with the Declaration of Helsinki and relevant laws and regulations, ensuring that the participants received the necessary protection and care and that the study findings were credible and reliable. Ethical approval was obtained from the Ethics Committee of the Inner Mongolia Nutrition Society (study no. A202207020). All participants provided written informed consent before the study began and agreed to consume a milk beverage during the trial.

### 2.2 Trial Design and Subject Recruitment

Participants were eligible if they were aged between 18 to 65 years and met the Rome III criteria for FD, with at least moderately severe symptoms (a score ≥ 4 for either epigastric pain syndrome (EPS) or postprandial distress syndrome (PDS) symptoms on the 7‐point global overall symptom scale) at the time of screening (Chey et al. 2019; Veldhuyzen van Zanten et al. 2006). All participants voluntarily agreed to comply with the study protocol and provided informed consent. The exclusion criteria were as follows: (1) a history or family history of colorectal cancer (CRC), celiac disease, or inflammatory bowel disease (IBD); (2) a confirmed diagnosis of organic intestinal diseases via previous colonoscopy; (3) subjects who had undergone major surgery in the past 2 months; (4) subjects with cow's milk protein allergy; (5) subjects who had taken antibiotics within the past 2 weeks; (6) subjects who had taken psychotropic drugs (e.g., anti‐anxiety and antidepressant medications) within the past 4 weeks; (7) subjects with a long‐term clinical need for probiotic supplementation; (8) subjects with a long‐term need for medications to alleviate constipation or diarrhea; (9) a confirmed diagnosis of severe diseases that the researchers deemed inappropriate for enrollment, such as myocardial infarction, cerebral infarction, and malignant tumors; (10) subjects with severe mental disorders who had poor behavioral control and were unable to cooperate with the study; and (11) illiterate subjects who could not understand the informed consent form or sign it independently. During the 28‐day trial, the participants consumed 200 mL of the assigned milk beverage daily. The EP group (experimental group) received a fermented milk beverage containing PC‐01, whereas the CP group (control group) received an active comparator, an acidified milk beverage (non‐fermented, without PC‐01). Throughout the trial, the participants were instructed not to consume any fermented food or probiotic products (e.g., kimchi, curd, yogurt, cheese, active milk beverages, probiotic solid drink, etc.) other than the study samples. PC‐01 fermented milk beverages and acidified milk beverages were supplied by China Mengniu Dairy Company Limited.

### 2.3 Randomization and Blinding

We referred to the sample size ranges in clinical trials of similar studies on the same disease (Bosch et al. 2026; Buckle et al. 2024; Cangemi et al. 2024; Liu, Tu, et al. 2025; Teh et al. 2021; Wang et al. 2024) and combined this with the difficulties in subject recruitment and compliance in the trial, as well as the practical experience and suggestions of clinical experts, to comprehensively determine the study protocol with a total sample size of approximately 60 cases. In addition, to collect more gut microbiome data, we adopted a 2:1 allocation ratio between the EP and CP groups.

A computer‐generated random number sequence was used to establish a random allocation sequence. The baseline characteristics of the participants were sent to the statistician via email, and the statistician assigned groups according to a pre‐specified random sequence. To ensure allocation concealment, a statistician encoded and concealed the group assignments. A double‐blind design was adopted; both the experimental and control products were packaged in sealed plastic containers with identical appearance, ensuring that neither the participants nor the investigators were aware of the group allocation until the end of the study. The active comparator, an acidified milk beverage, was acidified to an acidic pH using lactic acid. A difference test between the PC‐01 fermented milk beverage and the acidified milk beverage was performed in accordance with the ISO 4120:2021 Sensory analysis Methodology Triangle test (ISO. 2021) (*p* = 0.713) to ensure no significant sensory differences between the products in the EP and CP groups. During the trial, three patients in the EP group and two in the CP group withdrew due to their unwillingness to participate. Ultimately, 37 patients in the EP group and 18 in the CP group completed the study.

### 2.4 Sample Collection and Questionnaire Surveys

Gastrointestinal symptom severity and changes were assessed on days 0, 14, and 28 of the intervention using the 7‐point Global Overall Symptom Scale (GOSS) (Kamolsripat et al. 2024; Liu, He, et al. 2025) and the Gastrointestinal Symptom Rating Scale (GSRS) (Banihashem et al. 2023; Liang et al. 2025). The primary outcome was the GOSS score, and the secondary outcome was the GSRS score. All adverse events and safety outcomes were recorded throughout the intervention period. On days 0, 14, and 28, participants collected fecal samples at home using a sterile sampler provided in a fecal storage kit (Guangdong Longsee Biomedical Co. Ltd., Guangzhou, China). All samples were labeled, transported to the laboratory on dry ice, and stored at −80°C for subsequent analysis.

### 2.5 DNA Extraction and Shotgun Metagenomic Sequencing

Metagenomic DNA was extracted from the stool samples using the QIAamp Fast DNA Stool Mini Kit (Qiagen GmbH, Hilden, Germany) according to the manufacturer's instructions. The integrity of the isolated DNA was assessed using 1% agarose gel electrophoresis (Zhao et al. 2023). The purity and concentration of the DNA were evaluated using a Nanodrop spectrophotometer (260 nm/280 nm ratio) and the Qubit dsDNA Assay Kit in combination with a Qubit 2.0 fluorometer (Life Technologies, CA, USA), respectively. Finally, DNA samples with a concentration of > 20 ng/μL, purity OD260/280 of 1.8–2.0, and intact electrophoresis profiles were selected for library construction and sequencing (Wang et al. 2025). Libraries were prepared using the NEBNext Ultra DNA Library Prep Kit for Illumina (New England Biolabs, Ipswich, MA, USA) (Wang et al. 2023). The resulting libraries were sequenced on an Illumina NovaSeq platform (Illumina Novaseq 6000; Illumina Inc., San Diego, CA, USA) to generate paired‐end reads (Tianjin Novogene Technology Co. Ltd., Tianjin, China).

### 2.6 Bioinformatic Analysis

Raw sequencing reads were first assessed for quality using FastQC (Yang et al. 2022). Low‐quality reads were filtered out using KneaDdata, and any contaminating human sequences were removed by aligning the reads to the human genome using Bowtie software (Tu et al. 2024). Functional analyses of genes, modules, and pathways were performed using HUMAnN3 (Chen et al. 2021). MetaPhlAn3, a component of HUMAnN3, was used to profile the microbial community composition and calculate species abundance (Shumyatsky et al. 2022). These abundance profiles were integrated with the UniRef and MetaCyc databases for comparative analyses. Finally, the abundance of genes, metabolic pathways, and pathway coverage was quantified and statistically analyzed (Beghini et al. 2021).

### 2.7 Statistical Analyses

Statistical analyses and data visualization were performed in R software (v4.4.2) and Adobe Illustrator (v26.4.1). R packages (e.g., vegan, ggplot2, and ggpubr) were used to calculate the Shannon‐Wiener diversity index. Community structure was examined using Principal Coordinate Analysis (PCoA), and differences between the EP and CP groups were statistically tested using the Adonis test (permutational multivariate analysis of variance with 999 permutations). The nonparametric Wilcoxon and Mann–Whitney tests were used to statistically examine the differences in bacterial abundance and metabolic pathways between the EP and CP groups. A Spearman's correlation network between differential bacteria and metabolic pathways was constructed in Cytoscape (v3.5.1), including edges for medium‐strong correlations (*r* > 0.3 or r < −0.3, *p* < 0.05). Statistical significance was set at *p* < 0.05. According to the criteria reported by Bunchorntavakul et al. using the GOSS questionnaire, a clinical response (effective outcome) was defined as an improvement of > 50% in the GOSS score (Bunchorntavakul and Jaigla 2024). In the present study, the EP group was further subdivided into responders (EP‐R) and non‐responders‐ (EP‐NR). The Random Forest modeling was performed using the R randomForest package with the primary purpose of identifying the characteristic gut microbiota taxa at baseline (day 0) that could predict the clinical response of FD patients to the PC‐01 fermented milk beverage intervention The dataset was initially split into a training set (70%) and a validation set (30%) via stratified sampling to maintain balanced class distribution. Stratified 10‐fold cross‐validation was adopted to effectively mitigate the bias caused by artificial data partitioning. To ensure model stability and avoid overfitting, this study conducted 1000 bootstrap resampling iterations. For performance assessment, receiver operating characteristic (ROC) analysis was performed using the pROC package in R, and the area under the curve (AUC) was calculated to quantify the predictive accuracy of the model; an AUC of > 0.7 indicated favorable predictive performance.

## 3 Results

### 3.1 Baseline Characteristics of Participants

Table 1 presents the baseline demographic characteristics of participants. The male‐to‐female ratio was 14:23 in the EP group and 2:7 in the CP group. The mean body mass index (BMI) was 24.66 ± 6.45 in the EP group and 23.02 ± 3.24 in the CP group. The mean age was 30.81 ± 6.33 years in the EP group and 33.61 ± 9.24 years in the CP group. No significant differences were found between the groups in terms of age, sex distribution, BMI, or any other monitored baseline parameter (*p* > 0.05).

**TABLE 1:** Baseline characteristics of the participants.

Characteristic	Experimental group	Control group	*p*
*N*	37	18	
Gender, Male *n* (%): Female *n* (%)	14 (37.84%); 23 (62.16%)	4 (22.22%); 14 (77.78%)	0.26
Age, years	30.81 ± 6.33	33.61 ± 9.24	0.35
BMI, kg/m^2^	24.66 ± 6.45	23.02 ± 3.24	0.63
Current smokers [n (%)]	3 (7.50%)	0 (0%)	0.23
BMF	5.62 ± 2.56	6.44 ± 2.25	0.18
BSS	4.03 ± 1.26	4.22 ± 1.31	0.76
GOSS	21.89 ± 6.96	22.89 ± 10.92	0.8
GSRS	35.32 ± 10.45	37.17 ± 14.50	0.89

*Note:* *p*‐value: Wilcoxon test.

Abbreviations: BMI, body mass index (weight in kilograms divided by the square of the height in meters); BMF, bowel movement frequency, recorded as the frequency of bowel movements per week; BSS, bristol stool scale score; GOSS, 7‐point global overall symptom scale; GSRS, gastrointestinal symptom rating scale.

**FIGURE 1:**
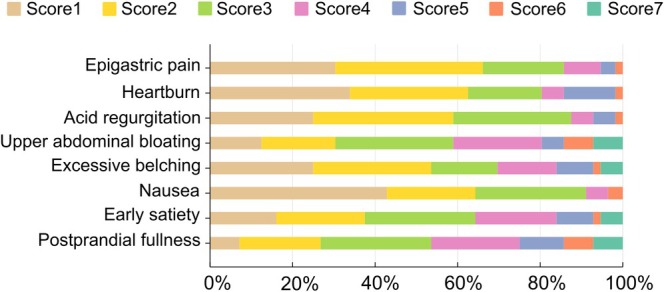
Percentage distribution of baseline scores for the 7‐point Global Overall Symptom Scale among participants.

The GOSS was used to assess FD symptom severity by evaluating eight symptoms: epigastric pain, heartburn, acid regurgitation, upper abdominal bloating, excessive belching, nausea, early satiety, and postprandial fullness. Each item was rated on a 7‐point Likert scale (1 = no symptoms; 7 = very severe symptoms). A score of 2 represents the lowest level of severity; thus, any item score ≥ 2 was considered indicative of the presence of that symptom. Among all patients in this study, 39 reported epigastric pain, 37 heartburn, 42 acid regurgitation, 49 upper abdominal bloating, 42 excessive belching, 32 nausea, 47 early satiety, and 52 postprandial fullness (Figure 1).

### 3.2 PC‐01 Fermented Milk Beverage Effectively Alleviated the Symptoms of Patients With FD

**FIGURE 2:**
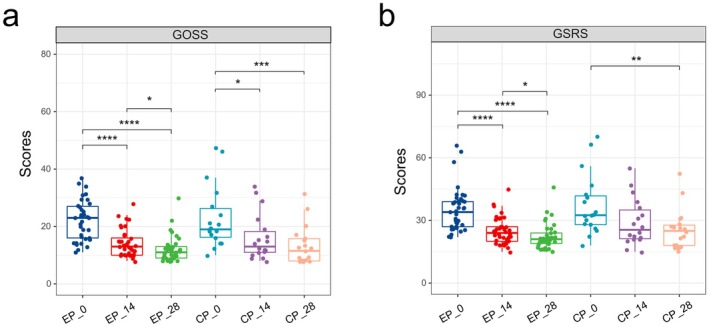
Intervention trial flow diagram and changes in questionnaire scores. (a) Changes in 7‐point global overall symptom scale (GOSS) scores in the two groups (b) Changes in gastrointestinal symptom rating scale (GSRS) scores in the two groups. The Wilcoxon test was used to evaluate the inter‐ and intragroup differences at the same and different time points, respectively; * *p* < 0.05, **p < 0.01, ***p < 0.001, and ****p < 0.0001. The prefix sample code represents the intervention (EP, experimental group; CP, control group), while the suffix sample code represents the time point (0, day 0; 14, after 14 days of intervention; 28, after 28 days of intervention).

Symptom severity was assessed using the GOSS and GSRS questionnaires. Both groups exhibited significant improvement in FD symptoms after the 28‐day intervention compared to baseline (*p* < 0.05, Figure 2, Table S1). Based on the criteria of Bunchorntavakul et al., the effective rate was 51.4% in the EP group, which was significantly higher than 22.2% in the CP group. A chi‐square test confirmed that the difference between the groups was statistically significant (*p* = 0.04). Given the exploratory, pilot nature of this study, the observed marginally significant difference suggests that the intake of PC‐01 fermented milk beverages may be associated with the improvement of FD symptoms.

### 3.3 PC‐01 Fermented Milk Beverage Affected the Gut Microbiota Composition of FD Patients

**FIGURE 3:**
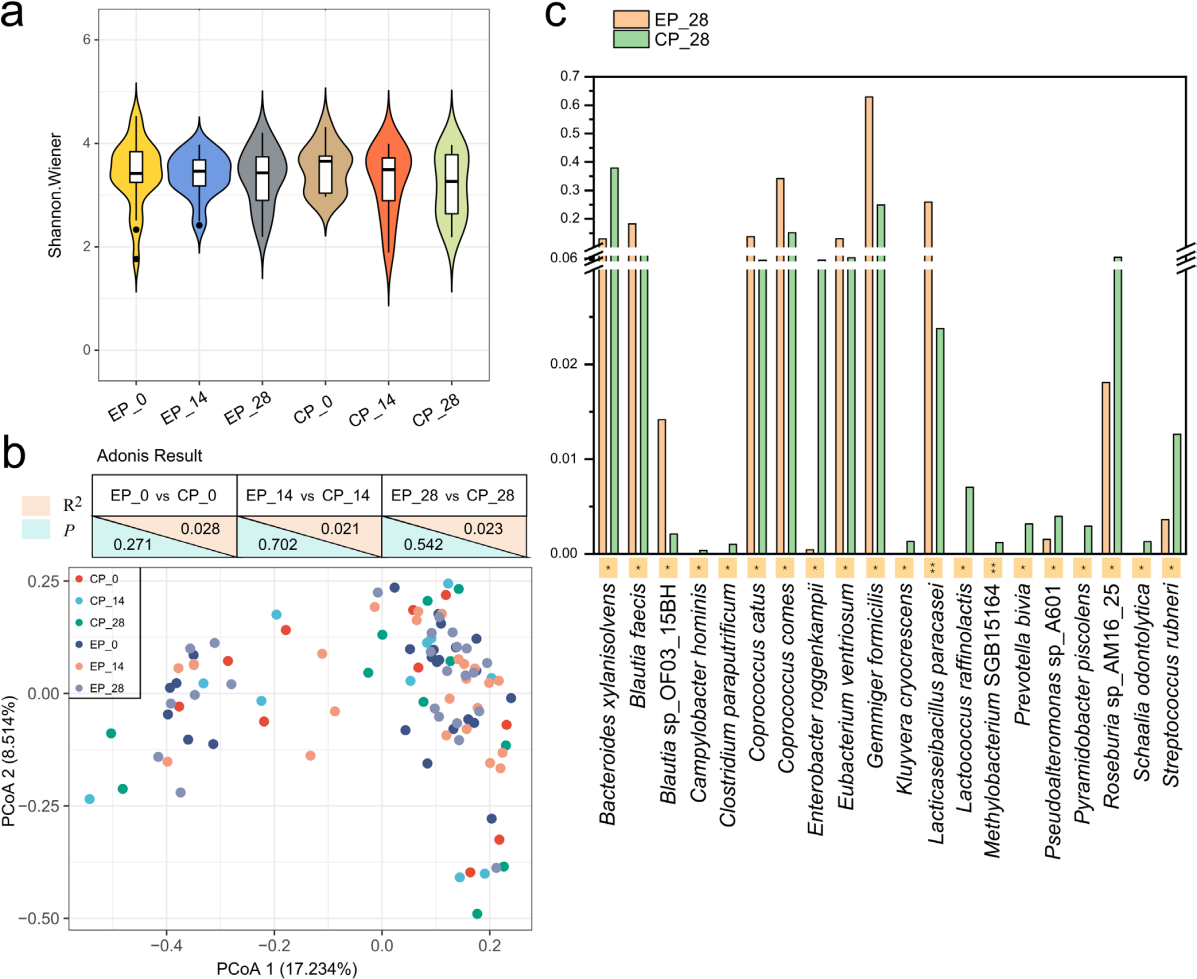
Microbial diversity and differentially abundant species in EP and CP groups. (a) Comparison of Shannon‐Wiener diversity index. (b) Comparison of beta diversity (assessed by principal coordinate analysis using Bray‐Curtis distance). Inter‐ and intragroup statistical differences at different time points were evaluated using the Adonis test. (c) Abundance of significant differential species in the EP and CP after the intervention (Wilcoxon test, *p* < 0.05). The prefix sample code represents the intervention (EP, experimental group; CP, control group), while the suffix sample code represents the time point (0, day 0; 14, after 14‐day intervention; 28, after 28‐day intervention). None of the identified species showed significant differences between the groups at baseline.

Accumulating evidence highlights the crucial role of the gut microbiota in the pathophysiology of FD, suggesting that alterations in the gut microbial community may contribute to the symptoms of FD. To explore this potential connection, we examined the effects of PC‐01 fermented milk beverages on the gut microbiota diversity and structure in patients with FD. Shannon‐Wiener diversity index was evaluated, and no significant changes were observed either within groups over time or between the EP and CP groups at any time point (*p* > 0.05, Figure 3a). PCoA based on Bray‐Curtis distances showed no clear separation between the EP and CP groups, a result corroborated by the Adonis test (Figure 3b). These findings indicate that the consumption of PC‐01 fermented milk beverages did not substantially alter the overall gut microbiota diversity or community structure. To assess compositional shifts in the microbiota after the intervention, we conducted a species‐level analysis (Figure 3c and Table S2). Comparison of fecal microbiota composition post‐intervention identified 20 taxa with significantly different abundances between the EP and CP groups (*p* < 0.05). Notably, the abundances of *L. paracasei, Blautia faecis*, *Coprococcus catus* ( *C. catus* ), *C. comes*, and *Gemmiger formicilis* ( *G. formicilis* ) were significantly higher in the EP group than in the CP group. In contrast, *Clostridium paraputrificum* and *Enterobacter roggenkampii* (*E. roggenkampii*) were more abundant in the CP group. Considering that the acidified milk beverage might also affect the gut microbiota, we separately investigated the taxa with significant differences before and after the intervention in both the EP and CP groups (Table S3). The relative abundance of *E. roggenkampii* significantly increased in the CP group after the intervention but showed the opposite trend in the EP group. *S. oralis* and *L. paracasei* were significantly enriched in the EP group, whereas *Klebsiella quasipneumoniae* (*K. quasipneumoniae*) and *K. variicola* were significantly decreased.

### 3.4 PC‐01 Fermented Milk Beverage Affected the Metabolic Pathways of FD Patients

**FIGURE 4:**
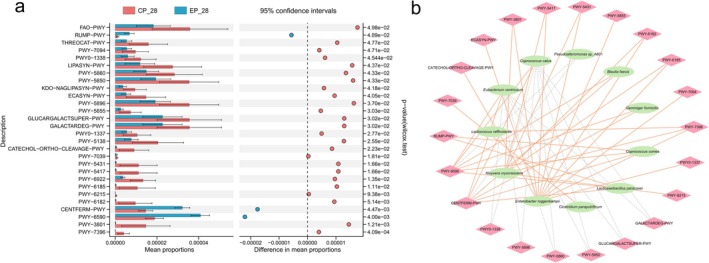
Differential metabolic pathways between the EP and CP groups after the intervention and the correlation network plot between the differential metabolic pathways and differentially abundant species. (a) Statistical differences in metabolic pathways were evaluated using the Mann–Whitney test at a confidence interval of 95%, *p* < 0.05. (b) Networks were constructed using the Spearman coefficient. Features with moderate to strong correlations are presented (*r* > 0.3 or r < −0.3, *p* < 0.05). Gray lines represent negative correlations, and orange lines represent positive ones. The prefix sample code represents the intervention (EP, experimental group; CP, control group), while the suffix sample code represents the time point (28, after 28‐days of intervention). None of the identified species showed significant differences between the groups at baseline.

Taxonomic analysis revealed differences in microbial composition between the two groups. To explore the functional potential of the gut microbiota, we annotated the metabolic pathways using HUMAnN3 and identified 520 distinct pathways. Of these, 28 metabolic pathways showed significant differences between the EP and CP groups after the intervention (Figure 4a and Table S4). These included five pathways related to carbohydrate metabolism, two related to aromatic compound synthesis and degradation, three related to amino acid metabolism, and six related to lipid metabolism. Notably, the pathways for pyruvate fermentation to butanoate (CENTFERM‐PWY) and the *Clostridium acetobutylicum* acidogenic superpathway (PWY‐6590) were significantly upregulated in the EP group (*p* < 0.05). In contrast, the pathways for catechol degradation to β‐ketoadipate (CATECHOL‐ORTHO‐CLEAVAGE‐PWY), catechol degradation (PWY‐5417), salicylic acid degradation (PWY‐6182), and fatty acid β‐oxidation (FAO‐PWY) were significantly downregulated in the EP group.

Spearman's correlation coefficient was used to explore the potential relationships between metabolic pathways and gut microbiota (Figure 4b). This analysis revealed that pyruvate fermentation to butanoate (CENTFERM‐PWY) and the superpathway of *Clostridium acetobutylicum* acidogenic (PWY‐6590) were significantly negatively correlated with *Pseudoalteromonas* sp_A601 and positively correlated with *Blautia faecis. E. roggenkampii* was significantly correlated with several degradation pathways, including 4‐methylcatechol degradation (PWY‐6185), catechol degradation to β‐ketoadipate (CATECHOL‐ORTHO‐CLEAVAGE‐PWY), superpathway of salicylate degradation (PWY‐6182), aromatic compounds degradation via β‐ketoadipate (PWY‐5431) and catechol degradation III (PWY‐5417). *L. paracasei* was significantly positively correlated with pyruvate fermentation to butanoate (CENTFERM‐PWY), superpathway of *Clostridium acetobutylicum* acidogenic (PWY‐6590) and formaldehyde oxidation I (RUMP‐PWY).

### 3.5 Random Forest Model Predicts the Specific Microbiota Characteristics of FD

**FIGURE 5:**
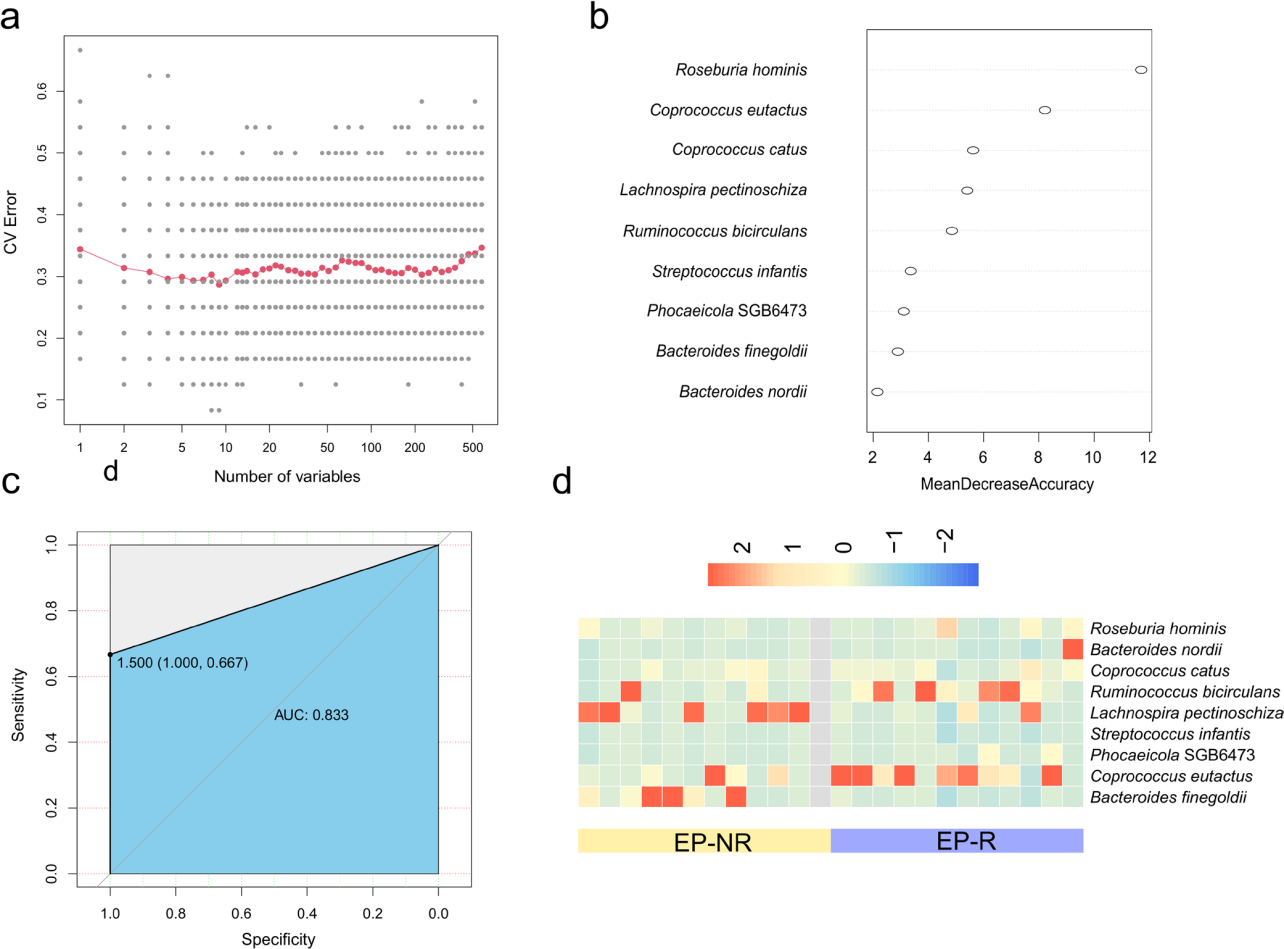
The potential characteristic bacteria screened and validated in the test set were selected as diagnostic markers for the efficacy of the PC‐01 fermented milk beverage. (a) Cumulative variation (CV) error curve. (b) Contribution degree of characteristic bacteria. (c) Receiver operating characteristic curves for the validation samples. (d) Heatmap showing the gut metagenomic classifier (the top nine characteristic bacteria for the symptom alleviation effect) between the EP‐R and EP‐NR groups at baseline (EP‐R, EP‐responsive; EP‐NR, EP‐unresponsive).

Since the response rate of the EP group was only 51.4% rather than 100%, it is suggested that there are individual differences in the therapeutic efficacy of the PC‐01 fermented milk beverage. Therefore, the participants in the EP group were classified as EP‐responsive (EP‐R) or EP‐unresponsive (EP‐NR) based on their clinical responses. A Random Forest classifier was then applied to distinguish between the EP‐R and EP‐NR subgroups using baseline microbiota data. Nine bacterial species with the highest predictive importance were identified via 10‐fold cross‐validation and used to build the final classification model (Figure 5a,b). The nine characteristic species identified *Roseburia hominis* , *Bacteroides nordii* , *C. catus* , *Ruminococcus bicirculans* (*R. bicirculans*), *Lachnospira pectinoschiza* , *S. infantis* , *Phocaeicola* SGB6473, *C. eutactus* , and *Bacteroides finegoldii*. The abundance of these nine species was compared between the EP‐R and EP‐NR groups (Figure 5d). *R. bicirculans* and *C. eutactus* were significantly more abundant in the EP‐R group, whereas *Lachnospira pectinoschiza* and *Bacteroides finegoldii* were enriched in the EP‐NR group. For model evaluation, the data were split into 70% training and 30% validation datasets, respectively. The model's performance on the validation set was assessed using the area under the receiver operating characteristic curve (AUC) of ROC, which yielded an AUC of 0.833 (Figure 5c). The results of this pilot study indicate that the baseline composition of the gut microbiota may be a key factor influencing the efficacy of PC‐01 fermented milk beverages.

## 4 Discussion

FD is a common gastrointestinal disorder that significantly impairs the quality of life and mental and physical health of patients owing to its chronic and recurrent nature (Cho et al. 2025). In this pilot study, we preliminarily explored the effects of PC‐01 fermented milk beverages on patients with FD. Consumption of the PC‐01 fermented milk beverage was associated with amelioration of FD‐related symptoms in the studied population, and this symptomatic improvement was found to be correlated with specific alterations in the relative abundance of gut microbiota taxa, implying a potential association between them.

To evaluate the potential efficacy of the PC‐01 fermented milk beverage, we assessed the severity of FD symptoms using both the GOSS and GSRS scoring systems. Both the EP and CP groups showed significant reductions in GOSS and GSRS scores after the intervention, suggesting that both the PC‐01 fermented milk beverage and the acidified milk beverage may ameliorate FD symptoms. However, the effective rate was significantly higher in the EP group (51.4%) than in the CP group (22.2%), indicating that the PC‐01 fermented milk beverage was more effective in alleviating FD symptoms in this pilot study. These results are consistent with those of previous studies. For example, one study reported that an *L. paracasei* LC‐37‐fortified milk beverage relieved FD symptoms, such as abdominal pain and belching (Sun et al. 2021). Similarly, yogurt containing *Lactobacillus gasseri* OLL2716 improved FD‐related symptoms (Ohtsu et al. 2017). Collectively, these findings suggest that probiotic‐fermented dairy products may be associated with alleviating FD symptoms. Therefore, probiotic‐enriched milk beverages may represent a potential non‐pharmacological adjunctive option for managing FD symptoms.

We also examined the effect of the PC‐01 fermented milk beverage on gut microbiota. No significant differences in α‐ or β‐diversity were observed between the EP and CP groups after the intervention, suggesting that the treatment did not markedly alter the overall community structure or ecological diversity of the microbiota. However, at a finer taxonomic resolution, 28 days of PC‐01 fermented milk beverage consumption significantly altered the relative abundance of specific taxa. The increase in *L. paracasei* abundance in the EP group suggests effective colonization of this probiotic strain in the human intestine. Research indicates that an increase in *Blautia* is beneficial to human health and plays a crucial role in maintaining intestinal environmental balance (Trischler et al. 2022). Its abundance is typically reduced in IBD, Crohn's disease (CD), and CRC (Liu et al. 2021). *G. formicilis*, a known butyrate‐producing bacterium, was also elevated in the EP group, and its abundance is typically depleted in patients with IBD (Ning et al. 2023). Butyrate is a key short‐chain fatty acid involved in maintaining intestinal health and immune regulation. *G. formicilis* is more abundant in healthy tissues, supporting its potentially beneficial role (Palmieri et al. 2021). *Coprococcus*, a core member of the healthy gut microbiota that ferments carbohydrates to produce butyrate (King et al. 2019), was also enriched in the EP group. A decrease in intestinal butyrate levels can increase luminal oxygen levels, impair epithelial barrier function, and facilitate pathogen overgrowth (Notting et al. 2023). Conversely, *Clostridium paraputrificum* was significantly depleted in the EP group, and this species is known to be enriched in patients with Crohn's disease (Sheikh et al. 2024). We found that *Campylobacter hominis* was also reduced in the EP group, and this bacterium has been reported to be more abundant in patients with CRC than in healthy individuals (Darnindro et al. 2025). Notably, although no significant differences were observed in either α‐ or β‐diversity between the EP and CP groups in this study, we identified several microbial taxa that differed significantly between the two groups. This finding suggests that the intervention primarily exerted targeted regulation on specific functional microbiota rather than causing drastic changes in the overall community diversity, which is consistent with previous reports (Dierikx et al. 2024; Grønbæk et al. 2025; Thomsen et al. 2024; Zhao, Li, et al. 2024). As comprehensive indices at the community level, α‐ and β‐diversities exhibit relatively low sensitivity to local variations in species abundance. Moreover, the gut microbiota possesses strong ecological stability; thus, the absence of significant differences in diversity does not indicate that the intervention was ineffective. A meta‐analysis indicated that therapeutic modulation of the gut microbiota does not require reversing large‐scale dysbiosis; instead, it may function by enhancing specific microbial functions (Lee et al. 2026). In addition, we found that the abundance of *E. roggenkampii* increased significantly in the CP group after the intervention, whereas the opposite effect was observed in the EP group. *E. roggenkampii* is an opportunistic pathogen that carries a variety of virulence factors and antibiotic resistance genes (Li and Sun 2024). Its increased abundance in the CP group may indicate a potential unhealthy disturbance in the intestinal microecology of these individuals, which may pose a risk of intestinal disease. The abundance of *S. oralis* was significantly increased in the EP group after intervention. Recent studies have revealed that this bacterium may interact with the host immune response and possess immunomodulatory properties (Zou et al. 2025). Meanwhile, the abundance of *K. quasipneumoniae* and *K. variicola* decreased significantly. Studies have indicated that these two emerging human pathogens are associated with infections in immunocompromised patients (Mishra et al. 2025; Sękowska et al. 2025). Therefore, the marked reduction in abundance in the EP group may reflect the effective inhibition of potential pathogenic bacteria by probiotic intervention. Overall, the PC‐01 fermented milk beverage intervention was associated with significant shifts in the relative abundance of specific gut microbiota taxa in FD patients. Compared with the CP group, the EP group exhibited increased abundance of putatively beneficial taxa (e.g., *C. catus and G. formicilis*) and decreased abundance of taxa potentially linked to intestinal disease progression (e.g., *Clostridium paraputrificum* and *Campylobacter hominis* ). These preliminary findings suggest that the alleviation of FD symptoms in the EP group may be mediated by these favorable changes in specific gut microbiota taxa; however, this potential mechanistic link requires further in‐depth verification.

We annotated gut microbial metabolic pathways to investigate how microbiota alterations affect host metabolism. Compared with the CP group, the EP group exhibited significant upregulation of pathways, such as formaldehyde oxidation I (RUMP‐PWY), whereas pathways, including fatty acid β‐oxidation I (FAO‐PWY) and ubiquinol‐7 biosynthesis (PWY‐5855), were downregulated. Formaldehyde oxidation I (RUMP‐PWY) was downregulated in patients with IBD (Schalich et al. 2024). Its suppression in the CP group of our study may similarly elevate oxidative stress, damage the intestinal mucosa, and impair gastrointestinal function, thereby contributing to the onset of FD‐related symptoms. In healthy colons, fatty acid β‐oxidation I (FAO‐PWY) and ubiquinol‐7 biosynthesis (PWY‐5855) are typically expressed at low levels (Palmieri et al. 2021). However, our findings of markedly increased expression of these pathways in the CP group suggest accelerated fatty acid metabolism. This metabolic shift can affect the contractile function of the intestinal smooth muscle and potentially trigger symptoms, such as abdominal distension and early satiety. We also observed that *Blautia faecis* abundance was significantly and positively correlated with pyruvate fermentation to butanoate (CENTFERM‐PWY) and the superpathway of *Clostridium acetobutylicum* acidogenic fermentation (PWY‐6590). *Blautia* ferments carbohydrates into short‐chain fatty acids (SCFAs) and is closely linked to immune system maturation, regulation of intestinal inflammation, and strengthening of the mucosal barrier (Fusco et al. 2023; Holmberg et al. 2024). The positive correlation between *Blautia faecis* and SCFA production pathways may be related to the improvement in FD‐related symptoms in the EP group, as SCFAs promote intestinal health and gastrointestinal motility (Martin‐Gallausiaux et al. 2021). Similarly, *E. roggenkampii* was significantly positively correlated with catechol degradation to β‐ketoadipate (CATECHOL‐ORTHO‐CLEAVAGE‐PWY) and catechol degradation III (PWY‐5417), consistent with the findings of Huang et al. (Huang et al. 2025). Although these catechol degradation pathways do not directly cause FD, the aberrant metabolism of aromatic compounds may indirectly affect gastrointestinal function by modulating gut‐brain neural signaling or altering microbial community balance. In summary, metabolic profiling suggests that PC‐01 fermented milk beverage intervention may be associated with a more balanced metabolic activity of the gut microbiota in patients with FD, potentially reducing the predicted activity of pathways that may exacerbate dyspeptic symptoms and enhancing the predicted activity of pathways (e.g., SCFA production) associated with intestinal health.

Despite these beneficial shifts, neither the pilot study nor previous trials achieved 100% symptom resolution, highlighting the considerable inter‐individual variability in probiotic response. This variability likely reflects the differences in baseline gut microbiota composition among the participants. Previous research has indicated that initial microbial diversity and functional potential can markedly influence individual responses to probiotic interventions (Zhao, Tie, Kwok, et al. 2024). In our pilot study, we found that the EP‐R group exhibited a higher baseline abundance of purportedly beneficial bacteria (e.g., *C. eutactus* ) than the EP‐NR group, whereas the EP‐NR group showed greater abundances of *Lachnospira pectinoschiza* and *Bacteroides finegoldii* . *C. eutactus* is a known SCFA producer (Valdes et al. 2024), while *Bacteroides finegoldii* is associated with elevated levels of trimethylamine N‐oxide (TMAO) (Yu et al. 2024). Additionally, *Lachnospira pectinoschiza* , which was more abundant in the EP‐NR group, has been reported to be closely linked to rectal neuroendocrine tumors (Gao et al. 2025), implying that its relatively high abundance in the EP‐NR group may signal a potentially less healthy intestinal microbial community in these non‐responder patients. These preliminary findings further underscore the potential close interrelationship between gut microbiota composition and host intestinal health and suggest that baseline microbial profiling may inform the development of personalized intervention strategies for patients with FD.

This study had some limitations. First, as a pilot study, the present research had a relatively small sample size and adopted a 2:1 randomization ratio between the EP and CP groups to obtain more comprehensive gut microbiota data. This limited the statistical power and generalizability of the findings, which require further validation in larger, multicenter studies. Second, owing to the lack of a fully inert placebo for the test product, an acidified milk beverage was used as the active comparator to minimize bias caused by sensory differences, which did not completely eliminate the potential biological effects. Third, the intervention period was relatively short (28 days), and no long‐term follow‐up data were available. Therefore, the long‐term durability of the observed effects cannot be determined. To address these limitations, future studies should be designed as large‐sample, multicenter, well‐balanced, randomized, double‐blind, controlled trials. The control formulation will be optimized, the intervention duration will be extended, and multiple follow‐up time points will be included to track the dynamic changes and long‐term sustainability of the intervention effects, thereby validating and expanding our preliminary findings.

Despite these limitations, the findings of this study are significant. As a pilot study, the present research preliminarily verified the feasibility of the PC‐01 fermented milk beverage in patients with FD and observed a positive trend in improvement. These findings provide preliminary data support and a feasibility basis for conducting large‐sample, long‐term randomized controlled trials in future studies.

## 5 Conclusion

Overall, the findings of the present pilot study indicate that intervention with the PC‐01 fermented milk beverage preliminarily reduced clinical scores and alleviated the symptoms of patients with FD. These beneficial effects are likely mediated by the modulation of the gut microbiota and metabolic pathways. These results preliminarily highlight the potential of PC‐01 fermented milk beverages as a safe and effective intervention for FD. Nevertheless, further long‐term studies with larger sample sizes are warranted to verify the therapeutic efficacy and elucidate the complex interactions among the gut microbiota, metabolites, and host intestinal function to provide effective strategies for the clinical management of FD.

## Author Contributions

**Xiangyang Zhang:** writing – original draft, formal analysis, visualization, validation, investigation. **Erna Sun:** writing – original draft, visualization, validation, investigation. **Xin Shen:** investigation. **Shusen Li:** supervision. **Zhixin Zhao:** investigation. **Qiuwen He:** supervision. **Heping Zhang:** supervision, project administration, conceptualization. **Yuejiao Wang:** supervision. **Julong Liu:** supervision. **Hongfeng Zhao:** supervision. **Feiyan Zhao:** writing – review and editing.

## Funding

This work was supported by National Natural Science Foundation of China (32394053, U22A20540), and the earmarked fund for CARS36.

## Conflicts of Interest

The authors declare no conflicts of interest.

## Supporting information


**Table S1:** Participant questionnaire score.

**Table S2:** The significant different species in the EP group and the CP group after the intervention with PC‐01 fermented milk beverage.

**Table S3:** The significant different species before and after the intervention in the EP group and the CP group.

**Table S4:** The significant different gut metabolic pathways in the EP group and the CP group after the intervention with PC‐01 fermented milk beverage.

## Data Availability

The data that support the findings of this study are openly available in National Genomics Data Cente at https://ngdc.cncb.ac.cn/, reference number GSA: CRA032519.

## References

[fsn371928-bib-0001] Abeltino, A. , D. Hatem , C. Serantoni , et al. 2024. “Unraveling the Gut Microbiota: Implications for Precision Nutrition and Personalized Medicine.” Nutrients 16: 3806. 10.3390/nu16223806. 39599593 PMC11597134

[fsn371928-bib-0002] Banihashem, S. S. , S. M. Mofatioshieh , R. Rastegar , and A. Sadeghi . 2023. “Comparing the Efficacy of Duloxetine and Nortriptyline in Alleviating the Symptoms of Functional Dyspepsia ‐ a Randomized Clinical Trial.” Frontiers in Psychiatry 14: 1297231. 10.3389/fpsyt.2023.1297231. 38293596 PMC10824943

[fsn371928-bib-0003] Beghini, F. , L. J. McIver , A. Blanco‐Míguez , et al. 2021. “Integrating Taxonomic, Functional, and Strain‐Level Profiling of Diverse Microbial Communities With bioBakery 3.” eLife 10: e65088. 10.7554/eLife.65088. 33944776 PMC8096432

[fsn371928-bib-0004] Bosch, D. , A. B. Beckers , J. T. W. Snijkers , et al. 2026. “Tailored Treatment of Functional Dyspepsia With Nortriptyline: A Multicenter, Double‐Blind, Placebo‐Controlled Trial.” Clinical Gastroenterology and Hepatology 56, no. 1. 10.1016/j.cgh.2026.01.013. 41616902

[fsn371928-bib-0005] Buckle, R. L. , L. C. Brown , and I. Aziz . 2024. “Randomized Trial in Postprandial Functional Dyspepsia: Reassurance and Diagnostic Explanation With or Without Traditional Dietary Advice.” Neurogastroenterology and Motility 36: e14733. 10.1111/nmo.14733. 38178367

[fsn371928-bib-0006] Bunchorntavakul, C. , and P. Jaigla . 2024. “Efficacy of Vonoprazan 10 and 20 mg for Patients With Proton Pump Inhibitor‐Refractory Functional Dyspepsia: A Double‐Blinded, Randomized Study.” JGH Open: An Open Access Journal of Gastroenterology and Hepatology 8: e70082. 10.1002/jgh3.70082. 39713745 PMC11659636

[fsn371928-bib-0007] Cangemi, D. J. , M. Montenegro , B. M. R. Spiegel , and A. B. E. Lacy . 2024. “Virtual Reality Improves Symptoms of Functional Dyspepsia: Results of a Randomized, Double‐Blind, Sham‐Controlled, Pilot Study.” American Journal of Gastroenterology 119: 210–213. 10.14309/ajg.0000000000002492. 37655713

[fsn371928-bib-0008] Chen, P. , Y. Guo , and L. Jia . 2021. “Interaction Between Functionally Activate Endometrial Microbiota and Host Gene Regulation in Endometrial Cancer.” Frontiers in Cell and Developmental Biology 9: 727286. 10.3389/fcell.2021.727286. 34631710 PMC8495019

[fsn371928-bib-0009] Chey, W. D. , B. E. Lacy , B. D. Cash , M. Epstein , P. E. Corsino , and S. M. Shah . 2019. “A Novel, Duodenal‐Release Formulation of a Combination of Caraway Oil and L‐Menthol for the Treatment of Functional Dyspepsia: A Randomized Controlled Trial.” Clinical and Translational Gastroenterology 10: e00021. 10.14309/ctg.0000000000000021. 30939487 PMC6493684

[fsn371928-bib-0010] Cho, N. , Y. Choi , H. Kim , et al. 2025. “Case Report: Improvement of Functional Dyspepsia Using Eight Constitution Acupuncture and Eight Constitution Diet ‐ A Report of Three Cases.” Frontiers in Medicine 12: 1545687. 10.3389/fmed.2025.1545687. 40771480 PMC12326475

[fsn371928-bib-0011] Darnindro, N. , M. Abdullah , N. Sukartini , et al. 2025. “Differences in Diversity and Composition of Mucosa‐Associated Colonic Microbiota in Colorectal Cancer and Non‐Colorectal Cancer in Indonesia.” World Journal of Gastroenterology 31: 100051. 10.3748/wjg.v31.i7.100051. 39991683 PMC11755252

[fsn371928-bib-0012] Dierikx, T. H. , A. M. Malinowska , J. Lukasik , et al. 2024. “Probiotics and Antibiotic‐Induced Microbial Aberrations in Children: A Secondary Analysis of a Randomized Clinical Trial.” JAMA Network Open 7: e2418129. 10.1001/jamanetworkopen.2024.18129. 38967929 PMC11227081

[fsn371928-bib-0013] Fusco, W. , M. B. Lorenzo , M. Cintoni , et al. 2023. “Short‐Chain Fatty‐Acid‐Producing Bacteria: Key Components of the Human Gut Microbiota.” Nutrients 15: 2211. 10.3390/nu15092211. 37432351 PMC10180739

[fsn371928-bib-0014] Gao, Y. , H. Zheng , M. Ye , et al. 2025. “Characteristics and Function of the Gut Microbiota in Patients With Rectal Neuroendocrine Tumors.” Journal of Cancer 16: 1040–1050. 10.7150/jca.103297. 39895797 PMC11786048

[fsn371928-bib-0015] Grønbæk, I. M. B. , S. I. Halkjær , S. Mollerup , et al. 2025. “The Effects of Probiotic Treatment With *Bifidobacterium breve* , Bif195 for Small Intestinal Crohn's Disease and the Gut Microbiome: Results From a Randomized, Double‐Blind, Placebo‐Controlled Trial.” Gut Pathogens 17: 19. 10.1186/s13099-025-00692-6. 40205497 PMC11984114

[fsn371928-bib-0016] Holmberg, S. M. , R. H. Feeney , P. K. V. Prasoodanan , et al. 2024. “The Gut Commensal Blautia Maintains Colonic Mucus Function Under Low‐Fiber Consumption Through Secretion of Short‐Chain Fatty Acids.” Nature Communications 15: 3502. 10.1038/s41467-024-47594-w. PMC11045866 38664378

[fsn371928-bib-0017] Huang, J. , B. Zhou , F. Zhu , et al. 2025. “Gut Microbiome Dysbiosis as a Potential Biomarker for Liver Metabolic Disorders in in Neonatal Hemolytic Jaundice.” BMC Pediatrics 25: 337. 10.1186/s12887-025-05692-8. 40301849 PMC12039124

[fsn371928-bib-0018] ISO . 2021. “Sensory Analysis‐Methodology‐Triangle Test”.

[fsn371928-bib-0019] Kailong, L. , K. Xiaohong , Z. Zhe , G. Shuai , and W. Jicheng . 2021. “Studies on Probiotic Properties and Safety of *Lactobacillus paracasei* PC‐01.” Journal of the Chinese Institute of Food Science and Technology 21: 47–52. 10.16429/j.1009-7848.2021.11.006.

[fsn371928-bib-0020] Kamolsripat, T. , N. Thinrungroj , K. Pinyopornpanish , et al. 2024. “Efficacy and Safety of Pinaverium Bromide as an Add‐On Therapy in Refractory Dyspepsia: A Randomized Controlled Trial.” JGH Open: An Open Access Journal of Gastroenterology and Hepatology 8: e13051. 10.1002/jgh3.13051. 38486875 PMC10938259

[fsn371928-bib-0021] King, C. H. , H. Desai , A. C. Sylvetsky , et al. 2019. “Baseline Human Gut Microbiota Profile in Healthy People and Standard Reporting Template.” PLoS One 14: e0206484. 10.1371/journal.pone.0206484. 31509535 PMC6738582

[fsn371928-bib-0022] Lee, K. , H. Kim , and J.‐H. Wang . 2026. “Clinical Evidence Linking the Gut Microbiome and Functional Dyspepsia: A Systematic Review and Meta‐Analysis.” Biomedicine 14: 457. 10.3390/biomedicines14020457. PMC12938209 41751356

[fsn371928-bib-0023] Li, Y. , and Z. Sun . 2024. “Phenotypic and Genomic Insights Into the Pathogenicity and Antimicrobial Resistance of an Enterobacter Roggenkampii Strain Isolated From Diseased Silver Arowana ( *Osteoglossum bicirrhosum* ).” Journal of Fish Diseases 47: e13898. 10.1111/jfd.13898. 38014710

[fsn371928-bib-0024] Liang, L. , H. Li , H. Doi , et al. 2025. “Integrated Effects of Kampo Treatment on Gastrointestinal Symptoms and Stress in Patients With Functional Dyspepsia: A Preliminary Prospective Observational Study.” Frontiers in Pharmacology 16: 1685656. 10.3389/fphar.2025.1685656. 41357899 PMC12678293

[fsn371928-bib-0025] Liu, C. H. , F. C. Tu , M. W. Wong , et al. 2025. “Efficacy of Jing Si Herbal Tea in Functional Dyspepsia: A Double‐Blind, Randomized, Placebo‐Controlled Study.” Journal of the Formosan Medical Association 124: 918–923. 10.1016/j.jfma.2024.09.008. 39256061

[fsn371928-bib-0026] Liu, J. , T. He , L. Ji , and L. Liu . 2025. “Low Fecal Elastase‐1: A Non‐Negligible Factor in Functional Dyspepsia Patients.” Digestive Diseases and Sciences 71: 1406–1415. 10.1007/s10620-025-09500-2. 41152608

[fsn371928-bib-0027] Liu, X. , B. Mao , J. Gu , et al. 2021. “Blautia‐a New Functional Genus With Potential Probiotic Properties?” Gut Microbes 13: 1–21. 10.1080/19490976.2021.1875796. PMC7872077 33525961

[fsn371928-bib-0028] Martin‐Gallausiaux, C. , L. Marinelli , H. M. Blottière , P. Larraufie , and N. Lapaque . 2021. “SCFA: Mechanisms and Functional Importance in the Gut.” Proceedings of the Nutrition Society 80: 37–49. 10.1017/s0029665120006916. 32238208

[fsn371928-bib-0029] Mishra, K. , T. Banerjee , G. Yadav , et al. 2025. “Emergence of Drug‐Resistant *Klebsiella pneumoniae* Phylogroups (K. Quasipneumoniae and *K. variicola* ) Causing Human Infections.” Microbiology Spectrum 13: e0019825. 10.1128/spectrum.00198-25. 40778769 PMC12403580

[fsn371928-bib-0030] Moreira, T. R. , D. Leonhardt , and S. R. Conde . 2017. “Influence of Drinking a Probiotic Fermented Milk Beverage Containing *bifidobacterium animalis* on the Symptoms of Constipation.” Arquivos de Gastroenterologia 54: 206–210. 10.1590/s0004-2803.201700000-27. 28591244

[fsn371928-bib-0031] Ning, L. , Y. L. Zhou , H. Sun , et al. 2023. “Microbiome and Metabolome Features in Inflammatory Bowel Disease via Multi‐Omics Integration Analyses Across Cohorts.” Nature Communications 14: 7135. 10.1038/s41467-023-42788-0. PMC10628233 37932270

[fsn371928-bib-0032] Notting, F. , W. Pirovano , W. Sybesma , and R. Kort . 2023. “The Butyrate‐Producing and Spore‐Forming Bacterial Genus Coprococcus as a Potential Biomarker for Neurological Disorders.” Gut Microbiome 4: e16. 10.1017/gmb.2023.14. 39295905 PMC11406416

[fsn371928-bib-0033] Ohtsu, T. , A. Takagi , N. Uemura , et al. 2017. “The Ameliorating Effect of *Lactobacillus gasseri* OLL2716 on Functional Dyspepsia in *Helicobacter pylori* ‐Uninfected Individuals: A Randomized Controlled Study.” Digestion 96: 92–102. 10.1159/000479000. 28768250 PMC5637312

[fsn371928-bib-0034] Ombasa, L. , J. Miller , L. Ware , et al. 2025. “Impact of Mānuka Honey on Symptoms and Quality of Life in Individuals With Functional Dyspepsia: Protocol for a Feasibility Randomized Controlled Trial.” JMIR Research Protocols 14: e66417. 10.2196/66417. 40397937 PMC12138293

[fsn371928-bib-0035] Palmieri, O. , S. Castellana , G. Biscaglia , et al. 2021. “Microbiome Analysis of Mucosal Ileoanal Pouch in Ulcerative Colitis Patients Revealed Impairment of the Pouches Immunometabolites.” Cells 10: 3243. 10.3390/cells10113243. 34831464 PMC8624401

[fsn371928-bib-0036] Qiu, J. J. , Z. Liu , P. Zhao , et al. 2017. “Gut Microbial Diversity Analysis Using Illumina Sequencing for Functional Dyspepsia With Liver Depression‐Spleen Deficiency Syndrome and the Interventional Xiaoyaosan in a Rat Model.” World Journal of Gastroenterology 23: 810–816. 10.3748/wjg.v23.i5.810. 28223725 PMC5296197

[fsn371928-bib-0037] Rai, A. K. , M. Yadav , R. K. Duary , and P. Shukla . 2025. “Gut Microbiota Modulation Through Dietary Approaches Targeting Better Health During Metabolic Disorders.” Molecular Nutrition & Food Research 69: e70033. 10.1002/mnfr.70033. 40195821

[fsn371928-bib-0038] Şanlier, N. , B. B. Gökcen , and A. C. Sezgin . 2019. “Health Benefits of Fermented Foods.” Critical Reviews in Food Science and Nutrition 59: 506–527. 10.1080/10408398.2017.1383355. 28945458

[fsn371928-bib-0039] Schalich, K. M. , M. A. Buendia , H. Kaur , et al. 2024. “A Human Milk Oligosaccharide Prevents Intestinal Inflammation in Adulthood via Modulating Gut Microbial Metabolism.” MBio 15: e0029824. 10.1128/mbio.00298-24. 38441000 PMC11005405

[fsn371928-bib-0040] Sękowska, A. , Y. Konechnyi , and A. Carrazco‐Montalvo . 2025. “Whole‐Genome Sequencing of Klebsiella Quasipneumoniae Subsp. Similipneumoniae Isolated From a Patient With Pneumonia.” Current Microbiology 82: 426. 10.1007/s00284-025-04414-8. 40745503 PMC12313781

[fsn371928-bib-0041] Sheikh, I. A. , J. Bianchi‐Smak , D. Laubitz , et al. 2024. “Transplant of Microbiota From Crohn's Disease Patients to Germ‐Free Mice Results in Colitis.” Gut Microbes 16: 2333483. 10.1080/19490976.2024.2333483. 38532703 PMC10978031

[fsn371928-bib-0042] Shumyatsky, G. , A. Burrell , H. Chaney , et al. 2022. “Using Metabolic Potential Within the Airway Microbiome as Predictors of Clinical State in Persons With Cystic Fibrosis.” Frontiers in Medicine 9: 1082125. 10.3389/fmed.2022.1082125. 36698799 PMC9868313

[fsn371928-bib-0043] Sun, E. , X. Zhang , Y. Zhao , et al. 2021. “Beverages Containing *Lactobacillus paracasei* LC‐37 Improved Functional Dyspepsia Through Regulation of the Intestinal Microbiota and Their Metabolites.” Journal of Dairy Science 104: 6389–6398. 10.3168/jds.2020-19882. 33714585

[fsn371928-bib-0044] Teh, K. K. , Y. K. Ng , K. Doshi , et al. 2021. “Mindfulness‐Based Cognitive Therapy in Functional Dyspepsia: A Pilot Randomized Trial.” Journal of Gastroenterology and Hepatology 36: 2058–2066. 10.1111/jgh.15389. 33373492

[fsn371928-bib-0045] Thomsen, M. , R. Vemuri , F. Huygens , S. Clarke , and L. Vitetta . 2024. “An Exploratory Study of a Multi‐Species Probiotic Formulation and Markers of Health in a Real‐World Oncological Cohort in the Time of Covid.” Inflammopharmacology 32: 2317–2335. 10.1007/s10787-024-01503-1. 38926298 PMC11300539

[fsn371928-bib-0046] Trischler, R. , J. Roth , M. T. Sorbara , X. Schlegel , and V. Müller . 2022. “A Functional Wood‐Ljungdahl Pathway Devoid of a Formate Dehydrogenase in the Gut Acetogens *Blautia wexlerae* , Blautia Luti and Beyond.” Environmental Microbiology 24: 3111–3123. 10.1111/1462-2920.16029. 35466558

[fsn371928-bib-0047] Tu, J. B. , W. J. Liao , S. P. Long , M. P. Li , and X. H. Gao . 2024. “Construction and Validation of a Machine Learning Model for the Diagnosis of Juvenile Idiopathic Arthritis Based on Fecal Microbiota.” Frontiers in Cellular and Infection Microbiology 14: 1371371. 10.3389/fcimb.2024.1371371. 38524178 PMC10957563

[fsn371928-bib-0048] Tziatzios, G. , E. Stylianakis , G. Damoraki , et al. 2025. “Third Generation Sequencing Analysis Detects Significant Differences in Duodenal Microbiome Composition Between Functional Dyspepsia Patients and Control Subjects.” Neurogastroenterology and Motility 37: e14955. 10.1111/nmo.14955. 39491051 PMC11650425

[fsn371928-bib-0049] Valdes, A. M. , P. Louca , A. Visconti , et al. 2024. “Vitamin A Carotenoids, but Not Retinoids, Mediate the Impact of a Healthy Diet on Gut Microbial Diversity.” BMC Medicine 22: 321. 10.1186/s12916-024-03543-4. 39113058 PMC11304618

[fsn371928-bib-0050] Veldhuyzen van Zanten, S. J. , N. Chiba , D. Armstrong , et al. 2006. “Validation of a 7‐Point Global Overall Symptom Scale to Measure the Severity of Dyspepsia Symptoms in Clinical Trials.” Alimentary Pharmacology and Therapeutics 23: 521–529. 10.1111/j.1365-2036.2006.02774.x. 16441473

[fsn371928-bib-0051] Wang, H. , C. Ma , Y. Li , et al. 2023. “Probio‐X Relieves Symptoms of Hyperlipidemia by Regulating Patients’ Gut Microbiome, Blood Lipid Metabolism, and Lifestyle Habits.” Microbiology Spectrum 11: e04440‐22. 10.1128/spectrum.04440-22. 37022264 PMC10269629

[fsn371928-bib-0052] Wang, J. , X. Bai , C. Peng , et al. 2020. “Fermented Milk Containing *Lactobacillus casei* Zhang and *Bifidobacterium animalis* Ssp. Lactis V9 Alleviated Constipation Symptoms Through Regulation of Intestinal Microbiota, Inflammation, and Metabolic Pathways.” Journal of Dairy Science 103: 11025–11038. 10.3168/jds.2020-18639. 33222846

[fsn371928-bib-0053] Wang, X. , M. A. Liu , J. He , et al. 2024. “Dietary Therapy of the Herbal Porridge Improves the Symptoms of Functional Dyspepsia: A Randomized, Double‐Blind, Placebo‐Controlled, Clinical Trial.” Food Science & Nutrition 12: 2104–2114. 10.1002/fsn3.3911. 38455174 PMC10916651

[fsn371928-bib-0054] Wang, X. , Z. Zhao , F. Zhao , et al. 2025. “Dual‐Omics Strategy for Selecting Optimal Fermentation Strains in Traditional Koumiss Production.” Food Chemistry: X 27: 102407. 10.1016/j.fochx.2025.102407. 40213331 PMC11984606

[fsn371928-bib-0055] XiaoRan, L. , L. ChenJian , T. XiaoDan , et al. 2020. “Gut Microbiota Alterations From Three‐Strain Yogurt Formulation Treatments in Slow‐Transit Constipation.” Canadian Journal of Infectious Diseases and Medical Microbiology 2020: 4583973. 32148595 10.1155/2020/4583973 PMC7049856

[fsn371928-bib-0056] Xv, Y. , J. Chen , and J. Lin . 2024. “Gut Microbiota and Functional Dyspepsia: A Two‐Sample Mendelian Randomization Study.” Frontiers in Microbiology 15: 1377392. 10.3389/fmicb.2024.1377392. 38881665 PMC11176457

[fsn371928-bib-0057] Yang, L. , M. Wang , X. Hu , et al. 2022. “EccDNA‐Oriented ITGB7 Expression in Breast Cancer.” Annals of Translational Medicine 10: 1344. 10.21037/atm-22-5716. 36660685 PMC9843317

[fsn371928-bib-0058] Yu, Y. , Y. Yin , J. Deng , X. Yang , S. Bai , and R. Yu . 2024. “Unveiling the Causal Effects of Gut Microbiome on Trimethylamine N‐Oxide: Evidence From Mendelian Randomization.” Frontiers in Microbiology 15: 1465455. 10.3389/fmicb.2024.1465455. 39526138 PMC11545679

[fsn371928-bib-0059] Zhang, Q. , G. Li , W. Zhao , et al. 2024. “Efficacy of *Bifidobacterium animalis* Subsp. Lactis BL‐99 in the Treatment of Functional Dyspepsia: A Randomized Placebo‐Controlled Clinical Trial.” Nature Communications 15: 227. 10.1038/s41467-023-44292-x. PMC10764899 38172093

[fsn371928-bib-0060] Zhao, F. , N. Tie , L. Y. Kwok , et al. 2024. “Baseline Gut Microbiome as a Predictive Biomarker of Response to Probiotic Adjuvant Treatment in Gout Management.” Pharmacological Research 209: 107445. 10.1016/j.phrs.2024.107445. 39396767

[fsn371928-bib-0061] Zhao, F. , Z. Zhao , D. Man , et al. 2023. “Changes in Gut Microbiota Structure and Function in Gout Patients.” Food Bioscience 54: 102912. 10.1016/j.fbio.2023.102912.

[fsn371928-bib-0062] Zhao, J. , H. Li , L. Y. Kwok , et al. 2024. “Improvement of Sleep Quality and Sub‐Health Conditions Through Pasteurized Fermented Milk Consumption: A Human Intervention Study.” Journal of Functional Foods 122: 106562. 10.1016/j.jff.2024.106562.

[fsn371928-bib-0063] Zhao, X. , X. Cheng , J. Ye , et al. 2024. “Efficacy and Safety of Zhishixiaopi Decoction in Functional Dyspepsia: A Meta‐Analysis of Randomized Controlled Trials.” PLoS One 19: e0301686. 10.1371/journal.pone.0301686. 38809916 PMC11135732

[fsn371928-bib-0064] Zhe, Z. , G. Shuai , Y. Xuejian , X. Liang , and Y. Su . 2021. “Identification and Biological Characteristics of *Lacticaseibacillus paracasei* PC‐01 and Lts Application in Live‐Bacteria Beverage.” Journal of Chinese Institute of Food Science and Technology 21: 265–272. 10.16429/j.1009-7848.2021.06.031.

[fsn371928-bib-0065] Zhong, L. , E. R. Shanahan , A. Raj , et al. 2017. “Dyspepsia and the Microbiome: Time to Focus on the Small Intestine.” Gut 66: 1168–1169. 10.1136/gutjnl-2016-312574. 27489239

[fsn371928-bib-0066] Zou, X. , H. Cao , L. Hong , et al. 2025. “Enrichment of *Streptococcus oralis* in Respiratory Microbiome Enhance Innate Immunity and Protects Against Influenza Infection.” Signal Transduction and Targeted Therapy 10: 272. 10.1038/s41392-025-02365-x. 40858544 PMC12381301

